# Cancer of the gallbladder—Chilean statistics

**DOI:** 10.3332/ecancer.2016.704

**Published:** 2016-12-21

**Authors:** Luis Villanueva Olivares

**Affiliations:** Arturo Lopez Foundation, Institute of Oncology, Chile Av Rancagua 878, Providencia, Santiago, 7500921, Chile; Clinical Hospital Arturo Lopez Perez Foundation, Clinical Hospital, University of Chile, Santos Dumont 999, Independencia, Santiago, 8380456, Chile

**Keywords:** gallbladder, biliary tract, Chile, epidemiology, obesity, Mapuche, ethnicity, cholelithiasis, socioeconomy, education, income

## Abstract

Chile has the world’s highest rate of incidence as well as death from cancer of the gallbladder and biliary ducts. The problem is most acute in the southern provinces. These areas constitute the low average income associated with low educational attainment and a high rate of obesity compared with the rest of Chile. We could also include genetic factors related to processes of lithogenesis to these elements which are more common among the Mapuche. This population sub-group could benefit from special government programmes for early diagnosis and treatment of lithiasic disease and for the management of risk factors such as obesity. In this way, we could reduce the mortality rate of gallbladder cancer.

## Introduction

Cancer of the gallbladder is one of the most common types of neoplastic disease among the Chilean population. It is associated with a high rate of mortality, and the net result is years of life lost owing to incapacity and premature death.

The demographic distribution within the national territory is heterogeneous and related to the socio-cultural and economic conditions of the different regions. It is also associated with the presence of risk factors in which cholelithiasis is the most important of all.

## Material

We reviewed data from the Department of Health Statistics and Information (DEIS) for the years 2000–2013 as well as data available in the First Cancer Register of Chile for 2003–2007 [1, 2]. We also reviewed data projected by the International Agency Research Cancer (IARC) on their page Globocan 2012 in order to compare the data available in Chile with other countries [3].

## Results

### The global picture of gallbladder cancer

For 2012, Globocan recorded a global rate of 178,801 new cases of gallbladder cancer and 142,823 deaths. The age-adjusted rate of incidence (ARI) was 2.2 for every 100,000 people, while the age-adjusted rate of mortality (ARM) was 1.7 for every 100,000 [3].

The greatest concentration of incidents and deaths occurs in the least developed regions of the world. Within these zones, the Western Pacific region shows the highest age-adjusted rate of incidence, which reaches 3.1 per 100,000 inhabitants, and an age-adjusted rate of mortality of 2.5 per 100,000 inhabitants.

Chile is the world leader, with incidence figures of 9.7 new cases for every 100,000 inhabitants each year, followed by Bolivia (8.1 per 100,000 inhabitants), South Korea (6.5 per 100,000 inhabitants), Laos (4.7 per 100,000 inhabitants), and Japan (4.7 per 100,000 inhabitants).

The ranking with respect to mortality figures is similar. Chile sits in first place with a ARM of 7.8 or 7.6, according to figures from DEIS [1], per 100,000 inhabitants, followed by Bolivia (7.5 per 100,000 inhabitants), South Korea (4.8 per 100,000 inhabitants), Laos (4.8 per 100,000 inhabitants), and Nepal (4.1 per 100,000 inhabitants) [3].

### The Chilean picture of gallbladder cancer

With respect to the incidences of gallbladder cancer in Chile, we only have local data gathered in a few population registers of cancer. These registers were begun in 1998 by health services in the regions of Antofagasta (Northern Chile) and Los Ríos (Southern Chile). These two registers along with those of Biobío province are integrated into the National Agency Research Cancer (IARC) database.

Drawing on these IARC population registers, the first report of Population Registers of Cancer in Chile was published in 2012, which included cases of incidence of the disease occurring between 2003–2007 [2].

The ARI of all cancers in Chile was 226.7 per every 100,000 men and 179.3 per every 100,000 women during that period.

Cancers presenting the highest ARI in men were: prostate (61.3 per 100,000), stomach (34.1 per 100,000), non-melanoma skin cancer (25.4 per 100,000), lung (19.7 per 100,000), colon (10.8 per 100,000), and gallbladder and biliary duct (8.7 per 100,000).

Cancers with the highest ARI for women were: breast (43.2 per 100,000), non-melanoma skin cancer (19.2 per 100,000), gallbladder and biliary duct (17.2 per 100,000), cervical (14.6 per 100,000), and stomach (12.8 per 100,000).

The rates of mortality for different cancers have undergone changes during the last decades.

According to the figures submitted by DEIS for the year 2000, the ARM for cancer was 118.7 deaths for every 100,000 inhabitants, while in 2013 the rate rose more than 20 points to 139.5 per 100,000 subjects. This meant an increase from 18,273 to 24,592 deaths.

**Figure 1.**
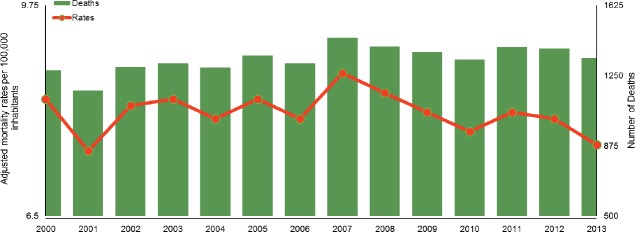
Age-adjusted mortality rate per 100,000 inhabitants and number of deaths of both sexes during the period 2000–2013 for gallbladder cancer (does not include malignant tumours of other parts and unspecified biliary tract tumours) according to data collected from the tables for the series of deaths and mortality because of malignant tumours according to age. Chile 1997–2013. DEIS (Department of Health Statistics and Information).

For the year 2000, 1,278 deaths related to gallbladder cancer were recorded (the rest of the biliary duct is not included in these statistics), which indicates an ARM of 8.3 per 100,000 inhabitants. These figures have remained constant during the period, amounting to 7.6 deaths for every 100,000 inhabitants in 2013 or 1343 deaths for that year (Figure 1).

In 2000, cancer of the gallbladder was the third most common neoplastic disease leading to death, after stomach and lung cancer. In 2013, it was in sixth place after stomach, lung, colon, rectal, prostate, and breast cancer (Table 1).

Among men, the ARM for gallbladder cancer is in eighth place for the year 2000 with 3.8 deaths for every 100,000 men. In 2013, there was a decline in rank to tenth place but a slight rise in the rate to 4.2 deaths per 100,000.

Among women, breast cancer was the most common cause of death from cancer in 2000, followed by stomach, and then gallbladder in third place with 12.7 deaths per 100,000 women. In 2013, breast cancer was the most common cause of death from cancer in women, while gallbladder cancer dropped to fourth place with an ARM of 11 deaths per 100,000.

**Figure 2.**
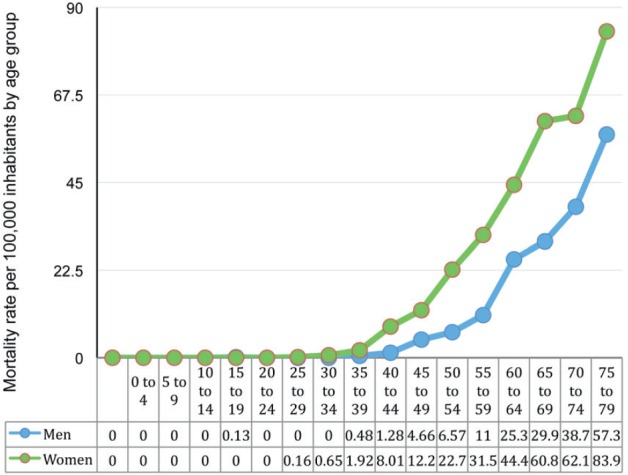
Age-adjusted mortality rate per 100,000 inhabitants by gender and age in 2013 for gallbladder cancer (does not include malignant tumours of other parts and unspecified biliary tract tumors) according to data collected from the tables for the series of deaths and mortality because of malignant tumours according to age. Chile. 1997–2013. DEIS.

The ARMs increase with age. For women, it increases after age 45, while it increases ten years later in men i.e. from age 55 (Figure 2).

### Regional picture of gallbladder cancer

For cancer of the gallbladder and biliary duct, the regions that show the highest rate of incidence in comparison with the rest of Chile include those from the O’Higgins region down to Aysén; that is, from the central south of the country to the south. Also included is the Coquimbo region located in the central north of the country. The highest rates are found in the regions of Los Lagos and Los Ríos (Southern Chile) [2].

**Figure 3.**
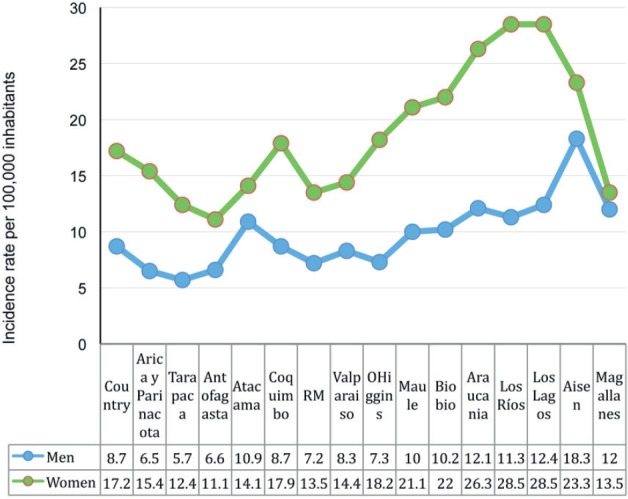
Age-adjusted and estimated incidence rate per 100,000 inhabitants by gender and regions of Chile from 2003–2007 for the gallbladder and bile duct cancer according to the First Report of Chilean Cancer Population Registries. 2003–2007. Department of Epidemiology. Ministry of Health.

For men, the regional picture for gallbladder and biliary duct cancer shows that the greatest number of incidents occur in the area from Maule to Magallanes (central south and south of the country) to which we can add the Atacama region. The highest rate was found in Aysén (Southern Chile) with 18.3 cases for every 100,000 men (Figure 3).

For women, cancer of the gallbladder and biliary duct shows high rates of incidence in the regions between and including O’Higgins to Aysén, to which we can add Coquimbo. The highest ARI was found in the regions of Los Lagos and Los Ríos, each with 28.5 per 100,000 women (Figure 3).

The mortality data for 2013 from DEIS show that the southern provinces had the highest rate of mortality for both men and women. The Los Ríos region showed the highest rate with 15.6 per 100,000 followed by the regions of Los Lagos, Aysén and Araucanía [1].

**Figure 4.**
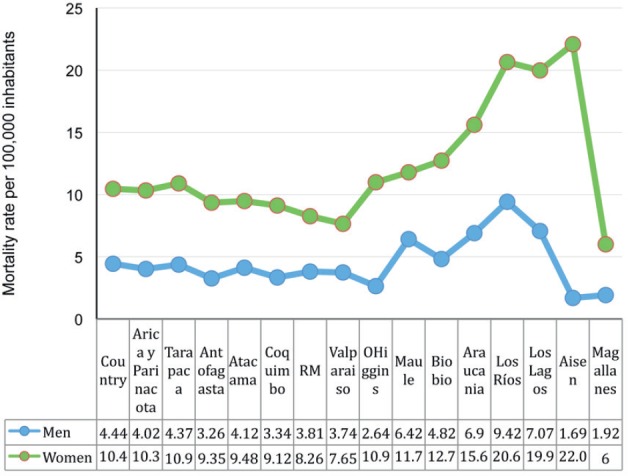
Age-adjusted mortality rate per 100,000 inhabitants by gender and regions of Chile in 2013 for gallbladder cancer (does not include malignant tumours of other parts and unspecified biliary tract tumours) according to data collected from the tables for the series of deaths and mortality because of tumours observed and adjusted by region. Chile. 2000–2013. DEIS.

For men, the province with the highest ARM is Los Ríos with 9.42, followed by Los Lagos with 7.07, and Araucanía with 6.9 per 100,000. For women, the highest rate is found in Aysén region with 22.09, followed by Los Ríos with 20.65, Los Lagos with 19.98, and Araucanía with 15.61 per 100,000 (Figure 4).

## Discussion

Chile has the greatest rate of incidence and mortality of gallbladder cancer worldwide. In order to explain this local reality, socioeconomic, environmental, and genetic factors that influence the development of the disease must be considered.

With regard to the socioeconomic and cultural reality that the disease can entail, a study of the population of Valdivia made between 1998 and 2000 evaluated the characteristics of the population, incidence rates and survival of 317 patients with gallbladder cancer. It showed that 64% of cases debuted in stage IV of the disease, and the incidence rates were higher in women with Mapuche ancestry particularly older than age 50 and with a low level of education (less than four years). These numbers rise to 269.2 new cases per 100,000 women [4].

In 2015, Herrera published an article in which he calculated the specific mortality from cancer adjusted for the educational level in the Chilean population. It showed that there is a higher rate of most of the cancers that are present in the population that had the lowest levels of education with the exception of breast cancer in women and lung cancer in men. These differences are even more pronounced in the case of gallbladder cancer in women with specific rates of mortality that are 49 times greater than those present in women with high levels of education. Therefore, mortality from cancer in Chile is strongly associated with the educational level of the population. This group could be more vulnerable to factors associated with a greater incidence of cancer such as exposure to higher risk conduct such as alcohol consumption, a lower level of physical activity, or a higher body mass index, consultation delayed to later stages of the disease or differences in timely access to treatment [5].

The regions in which the greatest incidences and mortalities are concentrated are also the poorest regions or those with the lowest income per capita. The regions with the lowest income, based on national account data provided by the central bank of Chile, were the Araucania, the Los Lagos and the Los Rios regions according to the number of inhabitants who live in them [6].

When comparing the survival rates of the patients who were treated in different healthcare establishments, whether in public hospitals or private ones, it was shown that the survival rate is lower in the public hospitals of the south versus public hospitals and private clinics in Santiago, Chile’s capital. However, by making an adjustment for the stage of the disease at the time of diagnosis, no differences were found in the survival rate between the different hospitals. From this, it is concluded that the only predictor of survival is the stage at which the disease presents at the time of the diagnosis. It is even more crucial than access to infrastructure and cutting-edge treatment technology. For this reason disease prevention takes on a greater importance than treatment techniques according to the study [7].

Obesity is another risk factor. This can contribute to the development of gallbladder cancer through its association with gallstones, its association with the increase of endogenous estrogens, or its association with fat cells which have ability to secrete a large amount of inflammatory mediators [8].

Chile is the country with the second highest rate of obesity in women (33.6%) after Venezuela and is third for men (24.5%) after Argentina and Venezuela. At the global level, Chile has a rate of prevalence near to or greater than even developed countries like the United States and much higher than the United Kingdom and other emerging countries like China [9].

The Chile National Health Survey 2009–2010 showed an obesity trend in accordance with educational level with a rate of 35.5% in the lower educational level and 18.5% in the higher. Women who live in rural areas also have a higher prevalence. The highest rate of prevalence of obesity is in the southern regions of Chile, especially the Los Rios region and the Aysen region which are regions in which the incidence and mortality from gallbladder cancer are higher than in the rest of the country [10].

Chilean studies have considered the population attributable fraction which is the percentage of cases that can be avoided by eliminating exposure to the risk factor like obesity as in this case. In the case of gallbladder cancer, this is 27% in women and 7% in men. Strategies that target reducing obesity envisage a significant reduction in the incidence of this type of cancer [9].

Gallbladder cancer is developed in the context of a chronic inflammation. In the majority of patients, the source of this chronic inflammation is cholesterol stones. The presence of stones increases the risk of gallbladder cancer four-to fivefold. Gallstones are reported in 79–100% of the gallbladder cancers [11].

In the Chilean population, gallstones have a high degree of prevalence of up to 26.7%, 36.6% in women and 16.6% in men [12]. Ethnic differences exist when comparing the Chilean general population with other ethnic groups that live in the same country such as the Rapa Nui ethnic group in which gallstone frequency is very low at 6.4% [13], whereas in the Mapuche population, it is so high that it reaches up to 35% [14]. Nevertheless, only 7% of the non-cholecystectomised population had symptoms compatible with gallstones, therefore it is possible to consider that most of the gallbladder pathology would appear asymptomatically This also becomes a problem for the early detection and surgical management of this condition which can lead to underestimating the need for resources to treat this disease. A coverage study of cholecystectomy in Chile showed that the populations that were subjected to more cholecystectomy surgeries were the Aysen and the Los Lagos regions, where there would be a greater prevalence of gallstones [12].

A lithogenic gradient exists between ethnic groups. This was demonstrated in a study of the mitochondrial DNA polymorphism of the Chilean population. An Amerindian maternal lineage was shown in the 100% of Mapuches and in 88% of the Hispanic Chileans. The same haplotypes were found in tribes in North and South America. The high prevalence of gallstones among non-obese young Mapuche people and Pima Indians suggests the presence of high penetrating lithogenic genes in these populations [14]. The most studied lithogenic polymorphisms correspond to the ABCG8-DH19 transporter gene in the ABCG5/G8 heterodimer pattern and that simultaneously could be related to a greater risk of developing cancer in certain ethnic populations like in the Chinese population [15]. These genes would participate in the generation of cholesterol stones, expressing abnormal molecular mechanisms in the secretion and/or solubility of cholesterol that would be present in women and is influenced by environmental factors like obesity [14].

The presence of gallstones is associated with inflammatory processes that could promote early changes in TP53 through increased cell turnover and oxidative stress. The deactivation of TP53 either by mutation or deletion is the most common genetic alteration in cancers and is observed in early stages of the natural history of gallbladder cancer, even in histologically normal epithelium. Exposure to environmental mutagens can also lead to alteration of TP53 as is the case with aflatoxins. Multiple polymorphisms in genes associated with the immune system, inflammation, and oxidative stress that have been linked to the development of gallbladder cancer like PTGS2, TLR2, TLR4, IL1RN, IL10, IL8, CCR5, LXRB, and OGG1. The use of some anti-inflammatories such as aspirin has been linked to a reduction in the risk of gallbladder cancer [15].

Other more unusual causes of chronic inflammation are primary sclerosing cholangitis, ulcerative colitis [16], chronic infection by Salmonella typhi and paratyphi [17], and infection by Helicobacter pylori [18] which could have a common endpoint in chronic injury of the large bile ducts with concentric periductal fibrosis, inflammation, and obliteration.

However, chronic inflammation of the gallbladder is only one part of the cause of the malignant transformation seen in gallbladder cancer. Many other factors have been identified such as the ingestion of certain medications, particularly oral contraceptives and methyldopa; exposure to certain chemical products, pesticides, rubbers, vinyl chloride; occupational exposure associated with work with textiles, petroleum, the paper industry, and the footwear industry. Added to these are exposure to pesticide-contaminated water, heavy metals, and radiation [8]. An association has also been found between gallbladder cancer and the aflatoxins present in grain-based agriculture as well as the ochratoxin A in red peppers. This association has been seen in the population of Amerindian ancestry in Chile that consumes them frequently as well as in the Peruvian and Bolivian population [19, 20, 21]

An increase in the incidence of gallbladder cancer is seen in certain hereditary syndromes such as Gardner syndrome, neurofibromatosis type I, and hereditary non-polypoid colon cancer.

## Conclusion

Identification of the group most susceptible to developing gallbladder cancer is an essential task in order to control the development of the disease, and also its incidence and mortality. One identified group is the descendants of Mapuche origin or ancestry, and they consitute mainly of women who live in the southern provinces of Chile. These population must be educated about the disease and given access to screening and treatment for lithiasic disease. Procedures must be started decades before the development of the cancer in order to detect the at-risk population and subject them to cholecystectomy. Chile’s programme of explicit health guarantees has included cholecystectomy for patients with gallstones between the ages of 35 and 49 since 2005. The impact of this intervention is likely to be observed in the next decade depending on the degree of resolution and adherence to the programme.

## Figures and Tables

**Table 1.** Age-adjusted mortality rate per 100,000 inhabitants of both sexes during the period 2000–2013 for the main malignant tumours, according to data collected from the tables for the series deaths and mortality by malignant tumours according to age. Chile. 1997–2013. DEIS.

	2000	2013
**Stomach**	19.6	18.4
**Lung**	12.3	17.2
**Gallbladder**	8.3	7.6
**Prostate**	8.3	11.6
**Breast**	6.7	8
**Colon**	5.6	9.3
**Rectum**	1.6	2.6
**Pancreas**	4.5	7.2
**Cervix**	4.4	3.2
**Liver and intrahepatic bile ducts**	4.1	6.3
**Total**	118.7	139.5
